# Renal function in Japanese HIV-1-positive patients who switch to tenofovir alafenamide fumarate after long-term tenofovir disoproxil fumarate: a single-center observational study

**DOI:** 10.1186/s12981-021-00420-5

**Published:** 2021-12-07

**Authors:** Kensuke Abe, Taku Obara, Satomi Kamio, Asahi Kondo, Junji Imamura, Tatsuya Goto, Toshihiro Ito, Hiroshi Sato, Nobuyuki Takahashi

**Affiliations:** 1 Division of Clinical Pharmacology and Therapeutics, Graduate School of Pharmaceutical Sciences, Tohoku University, Sendai, Japan; 2 Department of Pharmacy, National Hospital Organization Sendai Medical Center, 2-11-12 Miyagino, Miyagino-ku, Sendai, Miyagi 983-8520 Japan; 3 Department of Pharmaceutical Sciences, Tohoku University Hospital, Sendai, Japan; 4 Department of Infectious Diseases, National Hospital Organization Sendai Medical Center, Sendai, Japan; 5 JR Sendai Hospital, Sendai, Japan

**Keywords:** Tenofovir alafenamide fumarate, Tenofovir disoproxil fumarate, Renal function, eGFR, HIV

## Abstract

**Background:**

Tenofovir disoproxil fumarate (TDF) has a strong antiviral effect, but TDF is known to cause renal dysfunction. Therefore, we are investigating preventing renal dysfunction by replacing TDF with tenofovir alafenamide fumarate (TAF), which is known to be relatively safe to the kidneys. However, the changes in renal function under long-term use of TAF are not known. In this study, we evaluated renal function in Japanese HIV-1-positive patients switching to TAF after long-term treatment with TDF.

**Methods:**

A single-center observational study was conducted in Japanese HIV-1-positive patients. TDF was switched to TAF after at least 48 weeks of the treatment so we could evaluate the long-term use of TDF. The primary endpoint was the estimated glomerular filtration rate (eGFR) at 144 weeks of TAF administration. In addition, we predicted the factors that would lead to changes in eGFR after long-term use of TAF.

**Results:**

Of the 125 HIV-1-positive patients who were prescribed TAF at our hospital during the study period, 70 fulfilled the study criteria. The eGFR at the time of switching from TDF to TAF was 81.4 ± 21.1 mL/min/1.73 m^2^. eGFR improved significantly after 12 weeks of taking TAF but significantly decreased at 96 and 144 weeks. The factors significantly correlated with the decrease in eGFR at 144 weeks on TAF were eGFR and weight at the start of TAF.

**Conclusions:**

In this study, it was confirmed that switching to TAF was effective for Japanese HIV-1-positive patients who had been taking TDF for a long period of time and had a reduced eGFR. It was also found that the transition status depended on the eGFR and weight at the time of switch. Since HIV-1-positive patients in Japan are expected to continue taking TAF for a long time, renal function and body weight should be carefully monitored.

## Background

Tenofovir disoproxil fumarate (TDF), an anti-human immunodeficiency virus (HIV) and hepatitis B virus drug, has a strong antiviral effect. It is one of the recommended nucleoside reverse transcriptase inhibitors (NRTIs) in major guidelines such as those of the World Health Organization [1], Department of Health & Human Services [2] and European AIDS Clinical Society [3]. However, it is known that renal dysfunction is caused by the use of TDF [4]. In particular, Japanese HIV-1-infected patients with low body weight need to be carefully followed up [5]. Renal dysfunction due to TDF has been reported to be reversible, depending on the duration of treatment [6].

In addition, tubulointerstitial nephropathy, such as tubular necrosis, mitochondrial swelling, tubular atrophy, and interstitial fibrosis, may be observed [7]. The mechanism by which tubulointerstitial nephropathy develops is that tenofovir (TFV), the active ingredient of TDF, is taken up from the blood into tubular cells via organic anion transporter type 1 in the proximal tubule of the kidney, and then multidrug resistance protein type 4 excretes TFV in urine [8, 9]. During this process, TFV is enriched intracellularly, where it causes tubular cell damage [10].

Tenofovir alafenamide fumarate (TAF), which was approved and launched in 2016 in Japan, is said to have less effect on tubular cells than TDF [11]. TAF is highly stable in plasma, is metabolized to TFV after translocation into HIV target cells, and exerts an anti-HIV effect [12]. It shows strong antiviral effects at doses less than one-tenth those of TDF [13]. Therefore, it is expected that TAF may reduce the tubular injury and bone density decrease seen with TDF [11, 14]. The Japanese anti-HIV treatment guideline [15] has recommended TAF instead of TDF as one of the first-line treatments for NRTI since 2017.

We are investigating preventing renal dysfunction by switching TDF to TAF. There are reports that switching from TDF to TAF affects body weight and lipid metabolism [16]. In Japanese, increased body mass index (BMI) is associated with a decreased estimated glomerular filtration rate (eGFR) and chronic kidney disease [17, 18]. Therefore, even under the long-term administration of TAF, we need to pay close attention to some laboratory values. In this study, we evaluated the progression of renal function in Japanese HIV-1-positive patients 144 weeks after switching from long-term TDF to TAF. Furthermore, we investigated the status of weight and lipid metabolism in Japanese HIV-1-positive patients after switching from TDF to TAF.

## Methods

### Study design and patients

We performed a single-center observational study of Japanese HIV-1-positive patients using the medical records at the National Hospital Organization Sendai Medical Center in Sendai, a regional city in northern Japan.

In our hospital as of December 2019, 170 HIV-1-positive patients were on antiretroviral therapy. In this study, the subjects were Japanese HIV-1-positive adults aged 18 years or older of any sex who had changed from TDF 300 mg per day to TAF 25 mg or TAF 10 mg (the latter in the case of a regimen containing cobicistat or ritonavir) per day at our hospital before March 2020. Since the duration of the randomized, open-label, non-inferiority study (Study 934) comparing efavirenz (EFV) with emtricitabine (FTC) + TDF or zidovudine/lamivudine combination, which served as the approval review data for the FTC/TDF combination, was 48 weeks, to evaluate renal function after a certain period of TDF use, we defined 48 weeks or longer as long-term treatment and included only patients who had taken TDF for more than 48 weeks [19]. The subjects for drug change in this study were Japanese HIV-1-positive patients who were changed from TDF to TAF based on laboratory findings that serum creatinine remained above 1.2 mg/dL, which is the upper limit of the standard value in our hospital, or urinary β2-microglobulin (Uβ2MG) was abnormally high above 10,000 μg/L due to TDF administration, and Japanese HIV-1-positive patients who did not have obvious renal dysfunction but were explained by physicians and pharmacists that they would be changed from TDF to TAF to prevent deterioration of renal function by continued use of TDF. The third agent class of drugs such as non-nucleoside reverse transcriptase inhibitors (NNRTIs), protease inhibitors (PIs), and integrase strand transfer inhibitors (INSTIs), which were given with TDF, were not changed in order to monitor the impact of the switch from TDF to TAF.

In Japan, TDF was launched in April 2004. TAF was first launched in Japan in June 2016 as part of Genvoya® combination tablets. It was subsequently launched in December 2016 as part of the Descovy® LT combination tablet, which contains 10 mg of low-dose TAF for use in booster-containing antiretroviral therapy, and as part of the Descovy® HT combination tablet, which contains 25 mg of high-dose TAF for use in booster-free antiretroviral therapy.

This study was approved by the Clinical Research Department and the Human Research Ethics Committee of National Hospital Organization Sendai Medical Center and is registered under No. 31-93 and C31-86.

### Measurements

Laboratory values were studied at the start of taking TDF and when the patients were switched to TAF after taking TDF for more than 48 weeks. In addition, after the switch from TDF to TAF, laboratory tests were performed at 12, 24, and 48 weeks. After that, the tests were done every 48 weeks up to 144 weeks.

The laboratory tests measure the viral load of HIV-1 ribonucleic acid (HIV-1 RNA) and cluster of differentiation 4+ T cell (CD4) counts to determine the status of HIV infection suppression. In addition, eGFR, urine protein (UP), and blood urea nitrogen (BUN) are examined as indices of renal function, and Uβ2MG is used as an index of renal tubular disorder. BMI is used in the study as a measure of body weight to take into account the small stature of Japanese. Although triglyceride (TG), total cholesterol, HDL cholesterol, and LDL cholesterol are tested as lipid parameters in our hospital as an indicator of lipid metabolism, cholesterol-related tests were often not performed during the period of TDF administration, so we could not sufficiently tabulate the results. Therefore, TG is adopted as a lipid parameter in this study. In addition, patients taking hyperlipidemic drugs such as fibrates and statins were considered to have abnormal lipid metabolism. BMI was calculated from the height and the recorded body weight, and its classification was based on World Health Organization Western Pacific Region: BMI (kg/m^2^) = [body weight] × [height]^−2^ [20]. eGFR was calculated using eGFR_CG_ calculated by the Cockcroft–Goult equation and the eGFR recommended by the Japanese Society of Nephrology. eGFR_CG_ tends to be overestimated in the evaluation of renal function in Japanese [21], so the latter eGFR (mL/min/1.73 m^2^) = 194 × [serum creatinine]^−1.094^ × [age]^−0.287^ × [0.739 if female] [22] was adopted. The GFR classification to classify the stage of chronic kidney disease (CKD) was as follows: an eGFR greater than or equal to 90.00 mL/min/1.73 m^2^ at the start of TAF was defined as G1, G2 was eGFR greater than or equal to 60.00 mL/min/1.73 m^2^ and less than 90.00 mL/min/1.73 m^2^, G3a was eGFR greater than or equal to 45.00 mL/min/1.73 m^2^ and less than 60.00 mL/min/1.73 m^2^, G3b was eGFR greater than or equal to 30.00 mL/min/1.73 m^2^ and less than 45.00 mL/min/1.73 m^2^, G4 was eGFR greater than or equal to 15.00 mL/min/1.73 m^2^ and less than 30.00 mL/min/1.73 m^2^ and G5 was eGFR less than 15.00 mL/min/1.73 m^2^ [23].

The primary endpoint was the eGFR value after 144 weeks, using the time of switching from TDF to TAF as the baseline, to evaluate the effect of long-term use of TAF on renal function. As a secondary endpoint, eGFR values at 12, 24, 48, and 96 weeks after switching to TAF were compared with baseline. Changes in Uβ2MG, BMI, and TG values after switching to TAF were also checked as well as eGFR values and compared to baseline. Finally, we predicted the factors that would affect the change in eGFR after 144 weeks of prolonged use of TAF. eGFR, Uβ2MG, body weight, and TG values at the start of TAF, which are related to the endpoints of this study, were first discussed as factors that would affect the change in eGFR at 144 weeks. Other explanatory variables included age at the time of starting TAF, sex, duration of taking TDF, and the third agent class of drugs used in combination with TAF. The objective variable to assess the effect on eGFR after taking TAF was calculated as the difference in eGFR values from the start of taking TAF to 144 weeks, which was defined as a decrease of more than 10 mL/min/1.73 m^2^ [5].

### Statistical analysis

For eGFR, BMI, and TG, the baseline was set at the start of taking TAF, and statistical analysis was performed using the paired t-test for the mean values at each study period. For Uβ2MG, the median of each study period was statistically analyzed using the Wilcoxon signed rank test with the start of TAF as the baseline. For each group of eGFR and Uβ2MG after GFR classification, eGFR was analyzed by the paired t-test and Uβ2MG by the Wilcoxon signed rank test for each study period using the start of TAF as the baseline. For Uβ2MG after GFR classification, the median values at the time of switch to TAF were also statistically analyzed by Wilcoxon rank sum test for each group. The median TDF duration in each group after GFR classification was statistically analyzed by Wilcoxon rank sum test. To identify the factors affecting eGFR, multiple logistic regression analysis was performed. All statistical analyses were performed with JMP®, version 14.2 (SAS Institute, Cary, North Carolina, USA).

## Results

### Study population

Of the 125 HIV-1-positive patients who were prescribed TAF at our hospital during the study period, which ran until March 31, 2020, 70 patients fulfilled the inclusion criteria and constituted the study patients. The first set of excluded subjects were 5 patients who had taken TDF for less than 48 weeks before TAF, 13 patients who changed from abacavir, a non-Japanese person and 18 patients who took TAF without having taken TDF. Thus, the study started with 88 patients, but 11 patients were transferred to other hospitals during the course of the study, and 5 patients discontinued their visits. In addition, 2 patients did not receive TAF for the full 144 weeks.

The characteristics of the 70 patients in the study are shown in Table 1. The median age at the time of conversion from TDF to TAF was 44 (interquartile range = 37–49) years, 92.9% of the patients being male. Many of them had good viral control, with a median CD4 count of 480 (interquartile range = 332–627) cells/μL. The percentage of patients taking some third agent class drugs is shown in Table 1. The median duration of treatment with TDF was 274 (interquartile range = 128–454) weeks. The median serum creatinine at the time of the switch from TDF to TAF was 0.84 (interquartile range = 0.72–0.97) mg/dL, and the median eGFR was 80.89 mL/min/1.73 m^2^ (interquartile range = 68.12–92.02) mL/min/1.73 m^2^. The median Uβ2MG level was 267 (interquartile range = 114–869) μg/L, but one patient had an abnormally high level of up to 87,400 μg/L. Four patients (5.7%) were switched to TAF after the physician determined that they had TDF-related tubular damage based on their serum creatinine and Uβ2MG levels. They were all male. The other patients were switched from TDF to TAF prophylactically to avoid future renal damage. The median body weight was 70.1 (interquartile range = 60.8–75.9) kg, the median TG was 145 (interquartile range = 91–230) mg/dL, and patients receiving medication for hypertension, diabetes mellitus, and lipid metabolic disorders are shown in Table 1.

**Table 1:** Characteristics of patients

Variable	Baseline when switching to taking TAF from TDF
Number of patients	70
Median age, year (interquartile range)	44 (37–49)
Male, n (%)	65 (92.9)
Median HIV-1 RNA, copies/mL (interquartile range)	< 40 (< 40 to < 40)
Median CD4 counts, cells/μL (interquartile range)	480 (332–627)
Third agent class drugs	
INSTI, n (%)	61 (87.1)
Dolutegravir (DTG), n (%)	32 (45.7)
Elvitegravir (EVG), n (%)	18 (25.7)
Raltegravir (RAL), n (%)	11 (15.7)
PI, n (%)	9 (12.9)
Boosted darunavir (bDRV), n (%)	9 (12.9)
Median TDF duration, weeks (interquartile range)	274 (128–454)
Median serum creatinine, mg/dL (interquartile range)	0.84 (0.72–0.97)
Median eGFR, mL/min/1.73 m^2^ (interquartile range)	80.89 (68.12–92.02)
GFR categories, (mL/min/1.73 m^2^)^a^	
G1 (≧ 90), %	27.1
G2 (60–89), %	58.6
G3a (45–59), %	12.9
G3b (30–44), %	1.4
Median Uβ2MG, μg/L (interquartile range)	267 (114–869)
UP 1+ or 2+, %	6.0
Median BUN, mg/dL (interquartile range)	13.5 (11.0–16.0)
Median body weight, kg (interquartile range)	70.1 (60.8–75.9)
Median BMI, kg/m^2^ (interquartile range)	23.4 (21.3–26.1)
Median TG, mg/dL (interquartile range)	145 (91–230)
Hypertension, n (%)	10 (14.3)
Diabetes mellitus, n (%)	3 (4.3)
Abnormal lipid metabolism, n (%)	9 (12.9)
Fibrate treatment, n (%)	5 (7.1)^b^
Statin treatment, n (%)	4 (5.7)^b^

*TAF* tenofovir alafenamide fumarate, *TDF* tenofovir disoproxil fumarate, *HIV* human immunodeficiency virus, *RNA* ribonucleic acid, *CD4* cluster of differentiation 4+ T cell, *INSTI* integrase strand transfer inhibitor, *PI* protease inhibitor, *eGFR* estimated glomerular filtration rate, *GFR* glomerular filtration rate, *Uβ2MG* urinary β2-microglobulin, *UP* urine protein, *BUN* blood urea nitrogen, *BMI* body mass index, *TG* triglycerides

^a^No patients were categorized into G4 or G5

^b^No patient took these drugs together

### Change in renal function

**Fig. 1:**
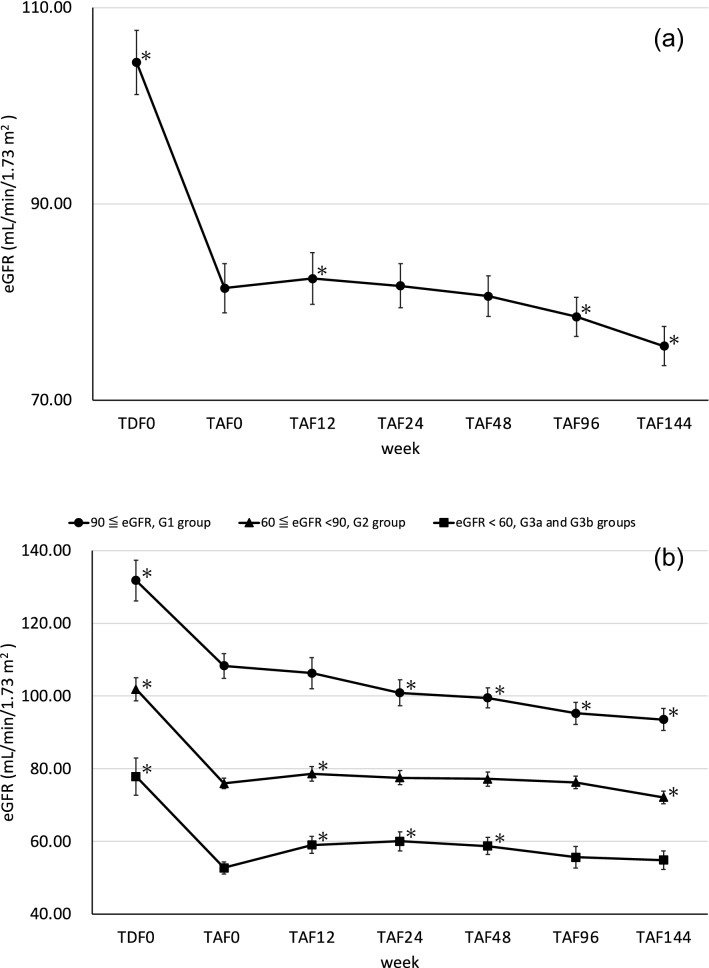
**A** Changes in eGFR (mean ± SE) of the patients taking TAF after taking TDF for more than 48 weeks. **B** Change in eGFR (mean ± SE) in patients taking TAF classified by eGFR values after taking TDF for more than 48 weeks. *eGFR* estimated glomerular filtration rate, *TDF* tenofovir disoproxil fumarate, *TAF* tenofovir alafenamide fumarate, *TDF0* start of TDF, *TAF0* start of TAF; TAF12, -24, -48, -96 and -144: 12 weeks, 24 weeks, 48 weeks, 96 weeks and 144 weeks after starting TAF. *p < 0.05 is defined as significant differences by the paired t-test

The mean eGFR ± standard error was 104.42 ± 24.60 mL/min/1.73 m^2^ at the time of starting antiretroviral therapy as shown in Fig. 1A. At the time of the change from TDF to TAF after more than 48 weeks of TDF medication, the mean eGFR ± standard error was 81.42 ± 21.10 mL/min/1.73 m^2^. Figure 1A shows a significant decrease in eGFR from TDF0 to TAF0 (mean difference = 24.07 mL/min/1.73 m^2^, 95% confidence interval = 19.45–28.68, p < 0.0001), which was significantly improved with TAF12 (mean difference = 2.49 mL/min/1.73 m^2^, 95% confidence interval = 0.38–4.60, p = 0.011). TAF96 (mean difference = − 2.92 mL/min/1.73 m^2^, 95% confidence interval = − 6.07–0.23, p = 0.034) and TAF144 (mean difference = − 5.79 mL/min/1.73 m^2^, 95% confidence interval = − 8.88 to − 2.70, p = 0.0002) showed a significant decrease compared to TAF0. The sample size for TAF0 is 70, while TDF0 is 56, TAF12 is 56, TAF24 is 64, TAF48 is 70, TAF96 is 70, and TAF144 is 69.

The trends of eGFR in the 3 groups based on GFR classification are shown in Fig. 1B. Incidentally, there were no G4 or G5 patients in the present study. According to the analysis by the Wilcoxon rank sum test, there was no difference between the mean TDF duration ± standard error of the G1 group (300 ± 34 weeks), the G2 group (287 ± 32 weeks) and the G3a and G3b groups (311 ± 60 weeks), G1 vs. G2 is mean difference = 7.74 weeks, 95% confidence interval = − 31.00–176.00, p = 0.11, G1 vs. G3a and G3b is mean difference = 1.53 weeks, 95% confidence interval = − 185.00–183.00, p = 0.65 and G2 vs. G3a and G3b is mean difference = − 4.79 weeks, 95% confidence interval = − 183.00–81.00, p = 0.36.

The eGFR in the G1 group decreased continuously after the switch from TDF to TAF. In particular, 24 weeks after switching to TAF, the eGFR decreased significantly (mean difference = − 7.07 mL/min/1.73 m^2^, 95% confidence interval = − 12.18 to − 1.97, p = 0.0048). Next, in the G2 group, eGFR increased significantly at 12 weeks after switching from TDF to TAF (mean difference = 2.75 mL/min/1.73 m^2^, 95% confidence interval = 0.39–5.12, p = 0.012). Thereafter, eGFR remained stable, without a significant difference, until 96 weeks (mean difference = 0.33 mL/min/1.73 m^2^, 95% confidence interval = − 2.11–2.78, p = 0.39), but at 144 weeks, eGFR decreased significantly (mean difference = − 3.52 mL/min/1.73 m^2^, 95% confidence interval = − 6.20 to − 0.85, p = 0.0056). Finally, in the G3a and G3b groups, eGFR was significantly higher at 12 weeks after switching from TDF to TAF compared to TAF0 (mean difference = 5.23 mL/min/1.73 m^2^, 95% confidence interval = 0.31–10.15, p = 0.020), and was also significantly higher at 24 (mean difference = 6.20 mL/min/1.73 m^2^, 95% confidence interval = 1.29–11.1, p = 0.0098) and 48 weeks (mean difference = 6.06 mL/min/1.73 m^2^, 95% confidence interval = 1.87–10.26, p = 0.0048) compared to TAF0. However, no significant difference occurred at 96 (mean difference = 2.92 mL/min/1.73 m^2^, 95% confidence interval = − 2.64–8.48, p = 0.13) and 144 weeks (mean difference = 2.16 mL/min/1.73 m^2^, 95% confidence interval = − 2.21–6.52, p = 0.15). In the G1 group, the number of samples for TAF0 is 19, while TDF0 is 12, TAF12 is 14, TAF24 is 18, TAF48 is 19, TAF96 is 19, and TAF144 is 19. In the G2 group, the number of samples for TAF0 is 41, while TDF0 is 35, TAF12 is 33, TAF24 is 37, TAF48 is 41, TAF96 is 41, and TAF144 is 40. In the G3a and G3b groups, the number of samples for TAF0 is 10, while TDF0 is 9, TAF12 is 9, TAF24 is 9, TAF48 is 10, TAF96 is 10, and TAF144 is 10.

**Fig. 2:**
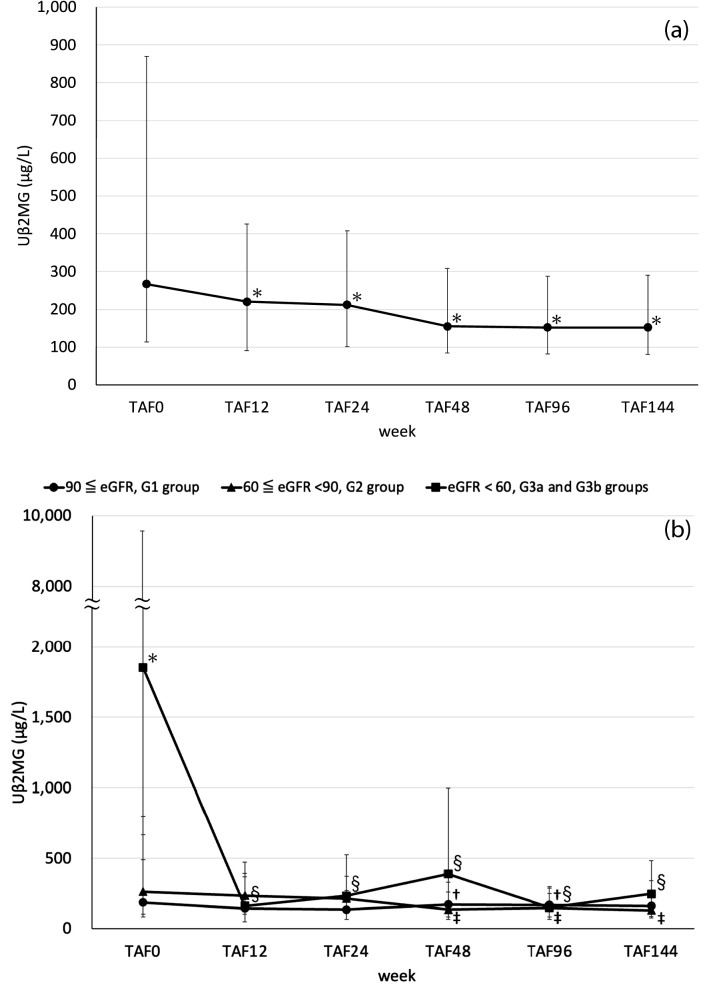
**A** Changes in Uβ2MG (median ± interquartile range: IR) of the patients switching from TDF to TAF. **B** Changes in Uβ2MG (median ± interquartile range: IR) of the patients switching from TDF to TAF. TAF: tenofovir alafenamide fumarate; TAF0: start of TAF; TAF12, -24, -48, -96 and -144: 12 weeks, 24 weeks, 48 weeks, 96 weeks and 144 weeks after starting TAF. *p < 0.05 is defined as a significant difference by the Wilcoxon rank sum test between groups. ^†^p < 0.05 is defined as a significant difference by Wilcoxon signed rank test for G1 group. ^‡^p < 0.05 is defined as significant differences by the Wilcoxon signed rank test for the G2 group. ^§^p < 0.05 is defined as significant differences by the Wilcoxon signed rank test for the G3a and G3b groups

As shown in Fig. 2A, Uβ2MG significantly decreased at TAF12 (mean difference = − 2600.8 μg/L, 95% confidence interval = − 6790.4–1588.8, p = 0.0092) compared with TAF0 and continued to significantly decrease until TAF144 (mean difference = − 2452.7 μg/L, 95% confidence interval = − 5641.6–736.2, p = 0.0011). The trends of UβMG in the 3 groups based on the GFR classification are shown in Fig. 2B. As in Fig. 1B, the groups were G1, G2, G3a and G3b. The UβMG of the G3a and G3b groups at the time of switching from TDF to TAF was significantly higher than that of the G1 and G2 groups. G1 vs. G2 is mean difference = 2.53 μg/L, 95% confidence interval = − 89.0–279.0, p = 0.57, G1 vs. G3a and G3b is mean difference = 7.91 μg/L, 95% confidence interval = 99.0–9792.0, p = 0.010 and G2 vs. G3a and G3b is mean difference = 11.46 μg/L, 95% confidence interval = 61.0–9108.0, p = 0.021. After the switch from TDF to TAF, UβMG in groups G3a and G3b decreased significantly at TAF12 (mean difference = − 16,514.0 μg/L, 95% confidence interval = − 52,967.0–19,938.3, p = 0.016), and the significant decrease continued thereafter until TAF144 (mean difference = − 15,441.0 μg/L, 95% confidence interval = − 45,004.0–14,121.2, p = 0.0078). In groups G1 and G2, UβMG, which was originally low at the time of switching from TDF to TAF, further decreased significantly at TAF48 (G1 mean difference = − 121.5 μg/L, 95% confidence interval = − 261.8–18.7, p = 0.021 and G2 mean difference = − 860.8 μg/L, 95% confidence interval = − 1980.2–258.6, p = 0.0038).

### Changes in BMI and TG

**Fig. 3:**
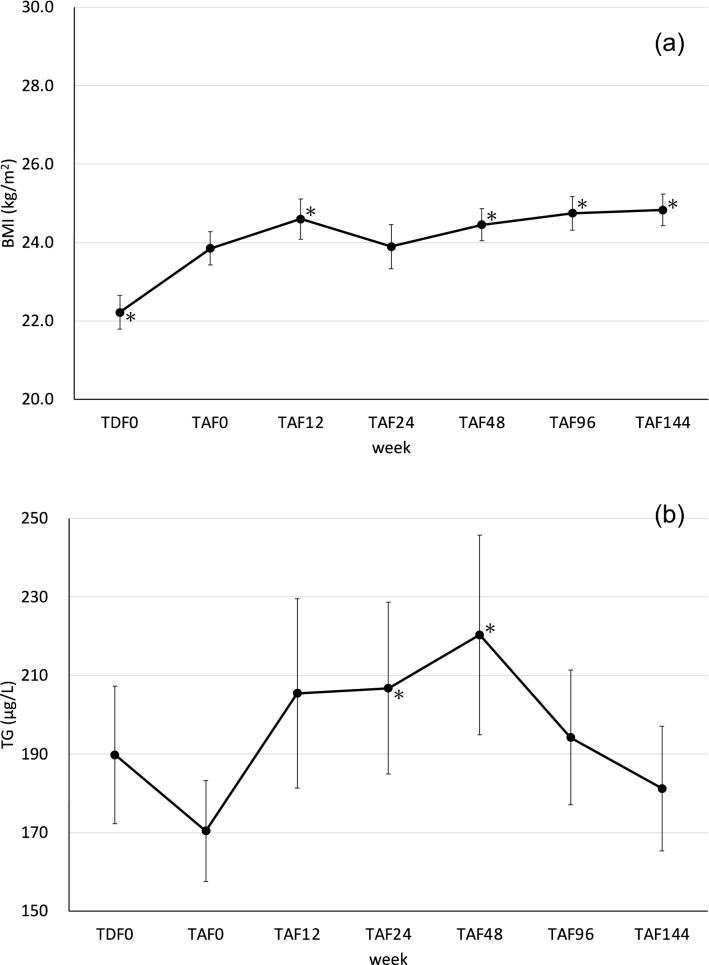
**A** Changes in BMI (mean ± SE) of the patients taking TAF after taking TDF for more than 48 weeks. **B** Changes in TG (mean ± SE) of the patients taking TAF after taking TDF for more than 48 weeks. TDF: tenofovir disoproxil fumarate; TAF: tenofovir alafenamide fumarate; TDF0: start of TDF; TAF0: start of TAF; TAF12, -24, -48, -96, and -144: 12 weeks, 24 weeks, 48 weeks, 96 weeks, and 144 weeks after starting TAF. *p < 0.05 is defined as a significant difference by the paired t-test

The changes in BMI are shown in Fig. 3A. The mean BMI increased significantly from 22.2 ± 0.4 kg/m^2^ to 23.8 ± 0.4 kg/m^2^ from TDF0 to TAF0 (mean difference = − 1.48 kg/m^2^, 95% confidence interval = − 2.16 to − 0.78, p < 0.0001). BMI continued to increase after the start of TAF treatment and increased even more significantly to 24.5 ± 0.4 kg/m^2^ at TAF48 (mean difference = 0.55 kg/m^2^, 95% confidence interval = 0.25–0.84, p = 0.0003). There was a significant increase in BMI at TAF 96 (mean difference = 0.82 kg/m^2^, 95% confidence interval = 0.39–1.25, p = 0.0002) and TAF 144 (mean difference = 0.90 kg/m^2^, 95% confidence interval = 0.50–1.30, p < 0.0001), but the mean BMI at TAF 144 was 24.8 ± 0.4 kg/m^2^, which was within the normal range for Japanese individuals [20]. Changes in TG are shown in Fig. 3B. During the period of taking TDF, the mean TG decreased from 190 ± 17 mg/dL to 170 ± 13 mg/dL after the start of TDF and up to TAF0 (mean difference = 6.0 μg/L, 95% confidence interval = − 34.3–46.3, p = 0.62). However, at week 48 after the switch from TDF to TAF, there was a significant increase in TG to 220 ± 25 mg/dL (mean difference = 49.9 μg/L, 95% confidence interval = 7.2–92.7, p = 0.011), whereas at TAF96 (mean difference = 25.3 μg/L, 95% confidence interval = − 8.0–58.7, p = 0.067) and TAF144 (mean difference = 10.8 μg/L, 95% confidence interval = − 21.0–42.6, p = 0.25), TG values decreased to the extent that they were not significantly different from those at TDF0.

### Factors associated with changes in eGFR from TAF0 to TAF144

Table 2 shows the predicted results of factors that affect the change in eGFR up to 144 weeks after switching from TDF to TAF. At 144 weeks after switching from taking TDF to TAF, the factors that showed a significant decrease of 10 mL/min/1.73 m^2^ or more in the difference in eGFR from TAF0 to TAF144 were eGFR (odds ratio = 1.15, 95% confidence interval = 1.06–1.25, p < 0.0001) and weight (odds ratio = 1.14, 95% confidence interval = 1.02–1.27, p = 0.007) at the start of TAF.

**Table 2:** Results of a logistic regression analysis to predict factors associated with a decrease in eGFR of more than 10 mL/min/1.73 m^2^ from TAF0 to TAF144

Variable	Odds ratio	95% confidence interval	p-value
eGFR	1.15	1.06–1.25	< *0.0001*
Uβ2MG	1.00	1.00–1.00	0.77
body weight	1.14	1.02–1.27	*0.01*
TG	0.99	0.99–1.00	0.25
Age	1.01	0.91–1.12	0.90
Sex, male	0.13	0.00054–30.03	0.45
TDF duration	0.32	0.0027–36.98	0.63
Third agent class drug, DTG	1.02	0.099–10.58	0.99

*eGFR* estimated glomerular filtration rate, *Uβ2MG* urinary β2-microglobulin, *TG* triglyceride, *TDF* tenofovir disoproxil fumarate, *DTG* dolutegravir

Variable at TAF0

Statistically significant p values are written in italics

## Discussion

In Japanese HIV-1-positive patients, a long time after switching from TDF to TAF, Uβ2MG significantly improved, but eGFR showed a significant decrease at 144 weeks. BMI increased moderately within the normal range. TG reached its highest value at 48 weeks but did not show a significant difference from the TDF0 or TAF0 value at 144 weeks.

Although patients in this study switched to TAF from the start of TDF, a pooled analysis of 26 trials by Gupta et al. compared renal function assessments between TAF-based and TDF-based regimens in virally suppressed HIV-infected patients at initiation of untreated antiretroviral therapy and at regimen change. The results pointed to a lesser decrease in median creatinine clearance in the TAF group compared to the TDF group (difference in least squares mean 6.0 mL/min, p ≤ 0.001 for week 96) [24].

In order to confirm the trend of eGFR in detail, the patients in this study were divided into three groups according to their eGFR values at the time of switching to TAF according to the published eGFR classification. In the G1 group, with high eGFR, eGFR continued to decrease after switching from TDF to TAF and had significantly decreased by 24 weeks. In the G2 group, with moderate eGFR, there was a temporary recovery of eGFR. In the G3a and G3b groups, with low eGFR, eGFR started increasing significantly 12 weeks after switching to TAF, and there was no significant decline from baseline by 144 weeks. In a prospective cohort study by Surial et al., by 18 months after switching from TDF to TAF, eGFR was associated with a decrease in eGFR of − 1.7 mL/min (95% confidence interval = − 2.7 to − 0.8) in patients with a baseline eGFR of 90 mL/min or greater and It was associated with an increase of 1.5 mL/min (95% confidence interval = 0.5–2.5) in patients with a baseline eGFR of 60 to 89 mL/min and 4.1 mL/min (95% confidence interval = 1.6–6.6) in patients with an eGFR of less than 60 mL/min [25]. Furthermore, Yoshino et al. reported the recovery of 3 groups of Japanese HIV-positive patients who had decreased eGFR due to taking TDF and discontinued TDF. Among them, the median value of eGFR at the time of the switch was higher in the group that showed a worsening of eGFR even after discontinuation of TDF than in the recovery group and the mild recovery group [6]. These findings are consistent with our present progress report.

TDF is known to cause tubular damage, and Uβ2MG is recommended as a test marker for tubular damage [26]. In our study, as shown in Fig. 2A, there was a significant decrease 12 weeks after switching from TDF to TAF, and the decrease continued thereafter, suggesting that tubular damage was improved. In addition, we investigated the course of Uβ2MG by GFR class, as shown in Fig. 2B. We found that Uβ2MG was higher only in the G3a and G3b groups, in which it was significantly higher than that in the G1 and G2 groups. Although the decrease in eGFR by TDF is related to tubular damage, we suggest that changing to TAF will improve tubular damage but not lead to a rapid recovery of eGFR, but a certain recovery can be achieved.

In summary, we believe that switching from TDF to TAF is effective in preventing the decline in eGFR and tubular damage in the low-eGFR group (eGFR less than 60 mL/min/1.73 m^2^). However, in the group with an eGFR of 60 mL/min/1.73 m^2^ or higher, the eGFR was significantly reduced by continuing to take TAF for a long period of time, though the extent of the decrease was not clear from the data in this study. We think it will be important to confirm the situation at 192 weeks and 240 weeks in the future.

In recent years, there have been many reports of weight gain and abnormal lipid metabolism associated with taking TAF [27–29]. In a report by Kuo et al. in Taiwanese HIV-positive individuals, another Asian ethnicity, significant weight gain and an increase in TG were observed at 48 weeks after switching from non-integrase inhibitor-based antiretroviral therapy to coformulated elvitegravir/cobicistat/emtricitabine/tenofovir alafenamide [30]. Therefore, in this study, we fixed the third agent class drugs and confirmed the changes in BMI and TG after switching from TDF to TAF. BMI showed a significant increase 12 weeks after switching from TDF to TAF but was within the normal range for Japanese people. TG was highest at TAF48 but declined thereafter and was not significantly different from baseline at TAF96 or TAF144. We believe that the reason for these findings is that information on weight gain and abnormalities in lipid metabolism caused by several anti-HIV drugs has been given to patients, and guidance on diet has been implemented during patient visits. Therefore, we consider that it is possible to prevent weight gain and abnormalities in lipid metabolism through an appropriate diet, even if the patient is taking TAF, but this is difficult to track and control in real-world settings.

To predict the factors associated with the change in eGFR after taking TAF, we performed logistic regression analysis using the variables shown in Table 2, with a decrease in eGFR difference of 10 mL/min/1.73 m^2^ or more from TAF0 to TAF144 as the objective variable. Turner et al. reported that the factor associated with the change in eGFR from before to after the switch from TDF to TAF was pre-switch eGFR [31]. However, in our long-term follow-up after switching from TDF to TAF, eGFR (p < 0.0001) and body weight (p = 0.01) at start of TAF were significantly associated with the objective variable. Kawamoto et al. [17] and Nomura et al. [18] reported that an increase in BMI was associated with a decrease in eGFR in Japanese patients with CKD. Although the subjects in this study were Japanese HIV-1-positive individuals without CKD, the association of body weight as a predictor of a decrease of more than 10 mL/min/1.73 m^2^ in the difference in eGFR from TAF0 to TAF144 is consistent with the results of previous studies. We address on the possibility that increased body weight due to the use of TAF may result in a decrease in eGFR.

This study was conducted at a single institution with a small sample size of only Japanese subjects, which are the main limitations of the study. However, this study design is a result of strict regulations and the elimination of missing survey items. We are the first to present the actual eGFR values of Japanese HIV-1-positive patients taking TDF for longer than 48 weeks and then continuing to take TAF for 144 weeks. We also detailed the course of eGFR trends up to 144 weeks after switching from TDF to TAF and predicted the factors affecting the difference from baseline after 144 weeks of taking TAF. This study also provides new details on other renal functions, such as the status of tubular damage as indicated by Uβ2MG, after the transition from baseline to 144 weeks of taking TAF. Furthermore, since there are few reports on BMI and TG after long-term use of TAF in Japanese individuals, these factors were also investigated.

Switching from TDF to TAF shows improvement in HIV-1-positive patients with impaired renal function, but continuous monitoring of renal function from all aspects is necessary for long-term use of TAF.

## Conclusions

In this study, it was confirmed that switching to TAF was effective for Japanese HIV-1-positive patients who had been taking TDF for a long period of time and had a reduced eGFR. It was also found that the transition status depended on the eGFR at the time of switch. Since HIV-1-positive patients in Japan are expected to continue taking TAF for a long time, renal function and body weight should be carefully monitored.

## Data Availability

All data generated or analyzed during this study are included in this published article.

## Abbreviations

TDF: Tenofovir disoproxil fumarate
HIV: Human immunodeficiency virus
NRTI: Nucleoside reverse transcriptase inhibitors
TFV: Tenofovir
TAF: Tenofovir alafenamide fumarate
BMI: Body mass index
eGFR: Estimated glomerular filtration rate
EFV: Efavirenz
FTC: Emtricitabine
Uβ2MG: Urinary β2-microglobulin
NNRTI: Non-nucleoside reverse transcriptase inhibitors
PI: Protease inhibitors
INSTI: Integrase strand transfer inhibitor
LT: Low dose tablet (10 mg)
HT: High dose tablet (25 mg)
HIV-1 RNA: Viral load of HIV-1 ribonucleic acid
CD4: Cluster of differentiation 4
UP: Urine protein
BUN: Blood urea nitrogen
TG: Triglyceride
HDL: High-density lipoprotein
LDL: Low-density lipoprotein
eGFR_CG_: Estimated glomerular filtration rate calculated using the Cockcroft–Gault equation
CKD: Chronic Kidney Disease
G1, G2, G3a, G3b, G4 and G5: GFR classification
TDF0: The time of starting antiretroviral therapy including TDF
TAF0: The time of change from TDF to TAF
TAF12, -24, -48, -96 and -144: 12 Weeks, 24 weeks, 48 weeks, 96 weeks and 144 weeks after starting TAF

## References

[1] World Health Organization. Interim guidelines on HIV/AIDS*.* 2018. https://apps.who.int/iris/bitstream/handle/10665/277395/WHO-CDS-HIV-18.51-eng.pdf. Revised on October, 2021.

[2] U.S. Department of Health & Human Services. Guidelines for the use of antiretroviral agents in adults and adolescents with HIV. 2021. https://clinicalinfo.hiv.gov/sites/default/files/guidelines/documents/AdultandAdolescentGL.pdf. Revised on October, 2021.

[3] European AIDS Clinical Society. Guidelines version 11.0. 2021. https://www.eacsociety.org/media/final2021eacsguidelinesv11.0_oct2021.pdf. Revised on October, 2021.

[4] Casado JL, del Rey JM, Bañón S (2016). Changes in kidney function and in the rate of tubular dysfunction after tenofovir withdrawal or continuation in HIV-infected patients. JAIDS.

[5] Nishijima T, Kawasaki Y, Tanaka N (2014). Long-term exposure to tenofovir continuously decrease renal function in HIV-1-infected patients with low body weight: results from 10 years of follow-up. AIDS.

[6] Yoshino M, Yagura H, Kushida H (2011). Assessing recovery of renal function after tenofovir disoproxil fumarate discontinuation. J Infect Chemother.

[7] Herlitz LC, Mohan S, Stokes MB (2010). Tenofovir nephrotoxicity: acute tubular necrosis with distinctive clinical, pathological, and mitochondrial abnormalities. Kidney Int.

[8] Ray AS, Cihlar T, Robinson KL (2006). Mechanism of active renal tubular efflux of tenofovir. Antimicrob Agents Chemother.

[9] Kohler JJ, Hosseini SH, Green E (2011). Tenofovir renal proximal tubular toxicity is regulated by OAT1 and MRP4 transporters. Lab Invest.

[10] Hall AM, Hendry BM, Nitsch D (2011). Tenofovir-associated kidney toxicity in HIV-infected patients: a review of the evidence. Am J Kidney Dis.

[11] Sax PE, Wohl D, Yin MT (2015). Tenofovir alafenamide versus tenofovir disoproxil fumarate, coformulated with elvitegravir, cobicistat, and emtricitabine, for initial treatment of HIV-1 infection: two randomised, double-blind, phase 3, non-inferiority trials. Lancet.

[12] Babusis D, Phan TK, Lee WA (2013). Mechanism for effective lymphoid cell and tissue loading following oral administration of nucleotide prodrug GS-7340. Mol Pharm.

[13] Ruane PJ, DeJesus E, Berger D (2013). Antiviral activity, safety, and pharmacokinetics/pharmacodynamics of tenofovir alafenamide as 10-day monotherapy in HIV-1-positive adults. JAIDS.

[14] Gallant JE, Daar ES, Raffi F (2016). Efficacy and safety of tenofovir alafenamide versus tenofovir disoproxil fumarate given as fixed-dose combinations containing emtricitabine as backbones for treatment of HIV-1 infection in virologically suppressed adults: a randomised, double-blind, active-controlled phase 3 trial. Lancet HIV.

[15] The Japanese Ministry of Health, Labour and Welfare. The guidelines for the treatment of HIV infection. 2021. https://www.haart-support.jp/guideline.htm. Revised on October, 2021.

[16] Schafer JJ, Sassa KN, O’Connor JR (2019). Changes in body mass index and atherosclerotic disease risk score after switching from tenofovir disoproxil fumarate to tenofovir alafenamide. Open Forum Infect Dis.

[17] Kawamoto R, Kohara K, Tabara Y (2008). An association between body mass index and estimated glomerular filtration rate. Hypertens Res.

[18] Nomura I, Kato J, Kitamura K (2009). Association between body mass index and chronic kidney disease: a population-based, cross-sectional study of a Japanese community. Vasc Health Risk Manag.

[19] Gallant JE, DeJesus E, Arribas JR (2006). Tenofovir DF, emtricitabine, and efavirenz vs. zidovudine, lamivudine, and efavirenz for HIV. N Engl J Med.

[20] World Health Organization Western Pacific Region. The Asia-Pacific perspective: redefining obesity and its treatment. 2000. https://apps.who.int/iris/handle/10665/206936. Revised on October, 2021.

[21] The Japanese Society of Nephrology. CKD treatment guide 2018. Tokyo Igaku-sha, 2018. https://cdn.jsn.or.jp/data/CKD2018.pdf. Revised on October, 2021.

[22] Matsuo S, Imai E, Horio M (2009). Revised equations for estimated GFR from serum creatinine in Japan. Am J Kidney Dis.

[23] National Kidney Foundation (2013). KDIGO 2012 clinical practice guideline for the evaluation and management of chronic kidney disease. Kidney Int Suppl.

[24] Gupta SK, Post FA, Arribas JR (2019). Renal safety of tenofovir alafenamide vs. tenofovir disoproxil fumarate: a pooled analysis of 26 clinical trials. AIDS.

[25] Surial B, Ledergerber B, Calmy A (2020). Changes in renal function after switching from TDF to TAF in HIV-infected individuals: a prospective cohort study. J Infect Dis.

[26] Nishijima T, Kurosawa T, Tanaka N (2016). Urinary β2 microglobulin can predict tenofovir disoproxil fumarate-related renal dysfunction in HIV-1-infected patients who initiate tenofovir disoproxil fumarate-containing antiretroviral therapy. AIDS.

[27] Venter WDF, Moorhouse M, Sokhela S (2019). Dolutegravir plus two different prodrugs of tenofovir to treat HIV. N Engl J Med.

[28] Surial B, Mugglin C, Calmy A (2021). Weight and metabolic changes after switching from tenofovir disoproxil fumarate to tenofovir alafenamide in people living with HIV: a cohort study. Ann Intern Med.

[29] Mallon PWG, Brunet L, Hsu RK (2021). Weight gain before and after switch from TDF to TAF in a U.S. cohort study. J Int AIDS Soc.

[30] Kuo PH, Sun HY, Chuang YC (2020). Weight gain and dyslipidemia among virally suppressed HIV-positive patients switching to co-formulated elvitegravir/cobicistat/emtricitabine/tenofovir alafenamide. Int J Infect Dis.

[31] Turner D, Drak D, O’Connor CC (2019). Renal function change after switching tenofovir disoproxil fumarate for tenofovir alafenamide in the HIV-positive patients of a metropolitan sexual health service. AIDS Res Ther.

